
JOURNAL INFORMATION
==============================
NLM Title Abbreviation: ScientificWorldJournal
Journal ID: ScientificWorldJournal
NLM Journal ID: 101131163
Journal ID: TSWJ

The Scientific World Journal

ISSN: 2356-6140
EISSN: 1537-744X
Publisher: Wiley

ARTICLE INFORMATION
==============================
PMCID: PMC6084378
PMID: 30147590
DOI: 10.1100/tsw.2001.125
Article ID: 507843
Article version: 1
Subjects: Short Report

Induced Heme Oxygenase-1 Upregulation Protects Pancreatic Beta Cells from Apoptosis In Vitro

Pileggi Antonello
Cattan Pierre
Molano R. Damaris
Berney Thierry
Vizzardelli Caterina
Fraker CHristopher
Oliver Robert
Ricordi Camillo
Pastori Ricardo
Bach Fritz H.
Inverardi Luca *
Diabetes Research Institute, University of Miami School of Medicine, USA
*Luca Inverardi: linverar@med.miami.edu
Publication date: 2001
Electronic publication date: 2001 Apr 4
Volume: 1
First page: 108
Last page: 108
Copyright: Copyright © 2001 Antonello Pileggi et al.
Copyright year: 2001
License: This is an open access article distributed under the Creative Commons Attribution License, which permits unrestricted use, distribution, and reproduction in any medium, provided the original work is properly cited.
License URL: https://creativecommons.org/licenses/by/3.0/

==============================
Miami *Nature Biotechnology* Short Reports
*TheScientificWorld* (2001) 1(S3), 108SR
ISSN 1532-2246; DOI 10.1100/TSW.2001.125

### INDUCED HEME OXYGENASE-1 UPREGULATION PROTECTS PANCREATIC BETA CELLS FROM APOPTOSIS IN VITRO

Antonello Pileggi, Pierre Cattan, R. Damaris Molano, Thierry Berney, Caterina Vizzardelli, Christopher Fraker, Robert Oliver, Camillo Ricordi, Ricardo Pastori, Fritz H. Bach, and Luca Inverardi*

Diabetes Research Institute, University of Miami School of Medicine
* linverar@med.miami.edu

**INTRODUCTION.** Transplantation of islets of Langerhans represents a viable therapeutic approach for the treatment of Type I diabetes mellitus (1). Unfortunately, transplanted islets are susceptible to allogeneic recognition and rejection, recurrence of autoimmunity and destruction by local inflammation at the site of implant. The latter phenomenon is characterized by microenvironment activation with production of proinflammatory cytokines (IL-1, IFN-γ, TNF-α) and oxygen radicals that might result in functional impairment and death of islet cells, and also contribute to amplifying the subsequent specific immune response (2,3). Islet β–cells are exquisitely susceptible to oxidative stress because of their insufficient anti-oxidant pool. Induction of islet cell protection against inflammation could therefore represent a viable strategy to improve overall graft fate. Heme oxygenase 1 (HO-1) has been described as an inducible stress protein capable of cytoprotection via radical scavenging and apoptosis prevention (4). The purpose of the present study was to analyze whether upregulation of HO-1 in a beta cell line (β-TC3) and in freshly isolated murine islets could result in protection from apoptosis.

**METHODS AND RESULTS.** HO-1 upregulation was reproducibly induced, in a dose dependent fashion, by incubating the beta cell line and isolated islets for 24 hours in the presence of iron protoporphyrin (FePP), as assessed by western blot and quantitative RT-PCR analysis. A 24-hr incubation in the presence or absence of FePP was performed in both Beta-TC3 cells and islets before induction of apoptosis. Subsequently, β-TC3 cells were incubated for 24-hrs in the presence of IL-1α (10U/ml) and IFN-γ (100U/ml) to induce the expression of Fas on their surface. A pro-apoptotic stimulus was then delivered via engagement of Fas by an agonistic anti-Fas antibody, followed by addition of protein G to induce cross-linking, and cells were cultured for 48-hrs before assay.

In freshly isolated murine islets, the pro-apoptotic signal was delivered by addition of TNF-α (5000U/ml) and cyclohexamide (CHX, 50 μg/ml) for additional 48 hours. Control islets were similarly treated in the presence or absence of FePP, without TNF-α and CHX. At the end of culture, β-TC3 and islet cell suspension were obtained by trypsinization, stained with propidium iodide, and analyzed by flow cytometry on a linear scale, considering apoptotic the cells within the distinct sub-$G_1$ peak, in which DNA condensation and fragmentation results in decreased staining. Analysis of apoptosis in the β-TC3 showed that the rate of apoptotic cells was inversely correlated to the concentration of FePP in the media, strongly suggesting a dosedependent protective effect of FePP-induced HO-1 upregulation. Protection in cells treated with 10, 40, 70, and 100μM of FePP was 6.1, 16.0, 18.8, and 29.1% respectively, when compared to control cells. Similarly, in murine islets FePP treatment resulted in a significant protection of islet cells from TNF-α-mediated apoptosis, when compared to non-treated cells (protection = 40.25±10.48%, $P<0.05$).

**DISCUSSION.** Our results suggest that FePP-induced HO-1 upregulation confer protection to islet cells from apoptosis induced via the Fas and the TNF-α pathways. Both Fas- and TNF-α apoptosis pathways play an important role in islet beta cell loss at the site of implant. Strategies aimed at inducing HO-1 upregulation might result in improved success in islet transplantation.

**REFERENCES.**

1. Shapiro, J.M., Lakey, J.R., Ryan, E.A., Korbutt, G.S., Toth, E., Warnock, G.L., Kneteman, N.M., and Rajotte, R.V. (2000) N. Engl. J. Med. 343, 230-238
2. Mandrup-Poulsen, T., Helqvist, S., Wogensen, L.D., Molvig, J., Pociot, F., Johannensen, J., and Nerup, J. (1990) Curr. Top. Microbiol. Immunol. 164, 169-193
3. Bottino, R., Fernandez, L.A., Ricordi, C., Lehmann, R., Tsan, M.F., Oliver, R., and Inverardi, L. (1998) Diabetes 47, 316-323
4. Petrache, I., Otterbein, L.E., Alam, J., Wiegand, G.W., and Choi, A.M. (2000) Am. J. Physiol. Lung. Cell Mol. Physiol. 278, L312-L319
